# Outcomes of Total Thyroidectomy in Large Goiters With Retrosternal Extension and Tracheal Compression: A Multivariate Analysis

**DOI:** 10.7759/cureus.73921

**Published:** 2024-11-18

**Authors:** Krithiga Sridar, SM Azeem Mohiyuddin, Sagayaraj A, Ravindra Deo, Kouser Mohammadi, Kalyani Raju, Sujatha Munireddy Papireddy

**Affiliations:** 1 Otorhinolaryngology and Head and Neck Surgery, Sri Devaraj Urs Medical College, Kolar, IND; 2 Pathology, Sri Devaraj Urs Medical College, Kolar, IND; 3 Anesthesia, Sri Devaraj Urs Medical College, Kolar, IND

**Keywords:** postoperative hypocalcemia, retrosternal extension of thyroid, total thyroidectomy, tracheal compression, tracheal deviation, tracheomalacia

## Abstract

Introduction: Large retrosternal goiters often cause tracheal compression and deviation, leading to respiratory symptoms and complicating surgical treatment. Total thyroidectomy is the treatment of choice though it carries a risk of complications due to the altered anatomy and its proximity to vital structures. This study examines the outcomes of total thyroidectomy in patients with retrosternal goiters and assesses the impact of tracheal compression on clinical results.

Methods: This retrospective study included 32 patients treated with total thyroidectomy for retrosternal goiter between January 2018 and June 2024. Patient records were analyzed for clinical presentation, tracheal status, extent of retrosternal extension, intubation details, surgery performed, intraoperative findings, and postoperative complications. Tracheal compression and deviation were noted by preoperative imaging and flexible fiberoptic bronchoscopy. A correlation between difficult intubation and tracheal status was attempted. Complications such as hypocalcemia, recurrent laryngeal nerve palsy, and tracheomalacia were assessed alongside surgical variables.

Results: Among the 32 patients, tracheal deviation was noted in 62.5% (n=20), and tracheal compression occurred in 43.8% (n=14). Complications included hypocalcemia in 43.8% (n=14) of cases, recurrent laryngeal nerve palsy in 6.25% (n=2), and tracheomalacia in 15.6% (n=5). Advanced airway management techniques such as flexible fiberoptic-assisted intubation were often required, particularly in cases with significant tracheal compression. Malignant goiters showed a higher incidence of tracheal deviation and postoperative complications but overall complication rates were comparable to benign cases.

Conclusion: Large retrosternal goiters carry the risk of airway compromise and surgical complications, particularly in the presence of tracheal compression. Hence, preoperative assessment of tracheal involvement and tailored surgical approaches are essential to manage airway complications and improve postoperative outcomes. A multidisciplinary approach is recommended for the management of these cases to enhance patient outcomes.

## Introduction

The term retrosternal goiter refers to an extension of the thyroid gland that descends below the thoracic inlet. The prevalence of retrosternal extension is about 1-20% of all goiters in various studies [1]. Nonsurgical treatment of retrosternal goiter with thyroid hormone or radioactive iodine ablation is almost always unsuccessful. Hence, total thyroidectomy remains the treatment of choice. Total thyroidectomy can be performed through a cervical incision in the neck, and in most cases, the gland can be safely delivered using sharp and blunt dissection. Purely retrosternal goiters may necessitate a sternotomy.

Large goiters with retrosternal extension have a greater tendency for compression of surrounding structures like the trachea which can cause various levels of respiratory distress. Even minor airway narrowing due to tracheal deviation and circumferential compression may significantly impact the flow of air. Long-term compression of the trachea may lead to postoperative collapse of the trachea and tracheomalacia leading to stridor, requiring reintubation or tracheostomy [2,3]. In patients with retrosternal goiters, challenges can arise at every stage of anesthesia management, including difficulties with bag-mask ventilation, laryngoscopy, and tracheal intubation due to tracheal compression or deviation. Postoperative complications like tracheomalacia can make ventilation difficult. Additionally, in critical situations where ventilation and intubation are not possible, surgical access to the trachea can be particularly challenging, adding complexity to airway management. 

The large size of the goiter has the tendency to alter the normal anatomy of the neck, thereby increasing the risk of injury to adjacent structures such as the recurrent laryngeal nerve. Surgery for retrosternal extension of goiter has a higher chance of devascularization of the parathyroids leading to hypocalcemia [4,5].

This study aims to document the outcomes of large goiters with retrosternal extension who underwent total thyroidectomy with or without central compartment clearance and/or mediastinal lymph node clearance, to evaluate its impact on tracheal compression and its outcomes. By analyzing the outcomes, this study aims to provide insights that can help refine surgical techniques and improve postoperative management strategies thereby reducing complications.

## Materials and methods

This was a retrospective study approved by the Central Ethics Committee, Sri Devaraj Urs Academy of Higher Education and Research (SDUAHER/KLR/R&D/CEC/S/PG/44/2024-25). Consent was taken from the patient/patient attenders to access patient records and to document the findings. The study included a total of 32 patients diagnosed with goiter having retrosternal extension who underwent total thyroidectomy with or without central compartment clearance and/or mediastinal lymph node clearance. The study period ranged from January 2018 to June 2024. Patients were selected based on inclusion and exclusion criteria.

Inclusion criteria

All patients aged between 18-65 years who had goiter with retrosternal extension and underwent total thyroidectomy with or without central compartment clearance and/or mediastinal lymph node clearance in the Department of Otorhinolaryngology and Head and Neck Surgery at a tertiary care rural hospital from January 2018 to June 2024 were included in the study.

Exclusion criteria

Patients who underwent complete total thyroidectomy following hemithyroidectomy done elsewhere and patients with parathyroid adenomas, hyperparathyroidism prior to surgery, second primary tumors, recurrent tumors, and skeletal abnormalities like scoliosis and kyphosis were excluded from this study.

Methodology

**Figure 1:**
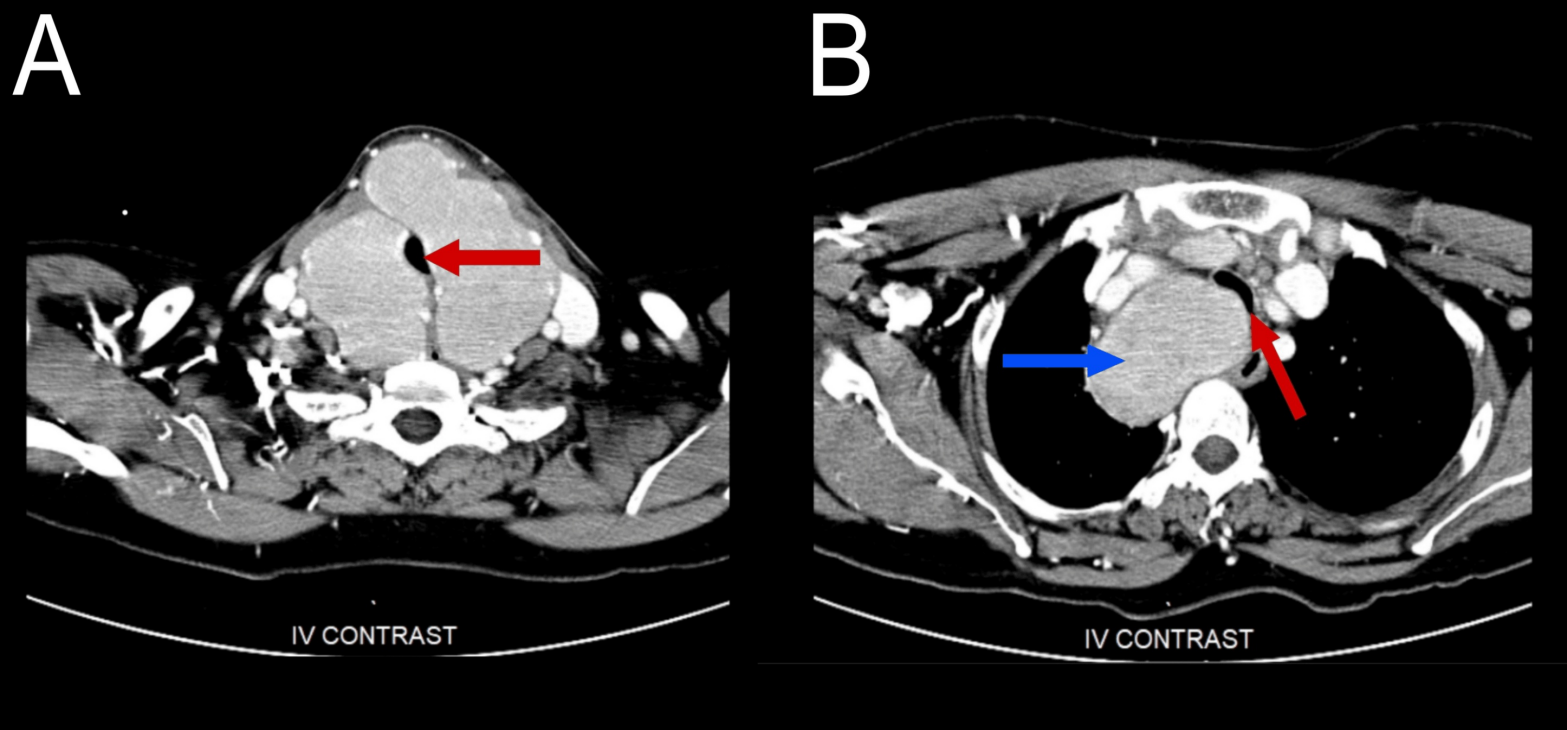
CECT images of a 47-year-old patiemt with multinodular goiter. Image A shows the enlarged thyroid gland compressing the trachea, causing luminal narrowing. Image B shows retrosternal extension of the enlarged thyroid gland with tracheal compression. The red arrow indicates tracheal compression and the blue arrow indicates the retrosternal extension of the enlarged thyroid gland. CECT: contrast-enhanced computed tomography.

The clinical data of 32 patients diagnosed with retrosternal goiter and who underwent total thyroidectomy was collected retrospectively from patient records. Demographic details, clinical presentation, and imaging findings were documented. Variables assessed included the extent of retrosternal extension, surgery performed, presence of lymph nodes, method of intubation, duration of surgery, and blood loss. Tracheal compression was characterized by a decrease in the size of the tracheal lumen, while tracheal deviation was characterized by a displacement of the trachea from the midline. The tracheal deviation was documented based on an X-ray neck. Tracheal compression was noted by CT scan and flexible fiberoptic bronchoscopy preoperatively (Figure 1). The tracheal status, including the presence of compression (anteroposterior or lateral) and to look for infiltration into the trachea and tracheal deviation was evaluated and correlated with clinical outcomes. In all cases, a preoperative assessment of vocal cord mobility was done. Postoperative complications such as recurrent laryngeal nerve palsy, hypocalcemia, and tracheomalacia were recorded. Tracheomalacia was suspected when softening of the tracheal rings was noted intraoperatively following removal of the thyroid gland. Hypocalcemia was considered when serum calcium levels were less than 8.5 mg/dl. The study also analyzed whether patients required prolonged ventilatory support due to tracheomalacia.

Statistical analysis

Data was entered into a Microsoft Excel (Microsoft Corp., Redmond, WA, USA) data sheet and was analyzed using IBM SPSS Statistics for Windows, version 22 (IBM Corp., Armonk, NY, USA). Categorical data was represented in the form of Frequencies and proportions. The Chi-square test was used as a test of significance for categorical data. An attempt was made whether there was a correlation between the extent of retrosternal extension and tracheal compression with difficulty in intubation. A p-value (probability that the result is true) of <0.05 was considered statistically significant after assuming all the rules of statistical tests.

## Results

**Figure 2:**
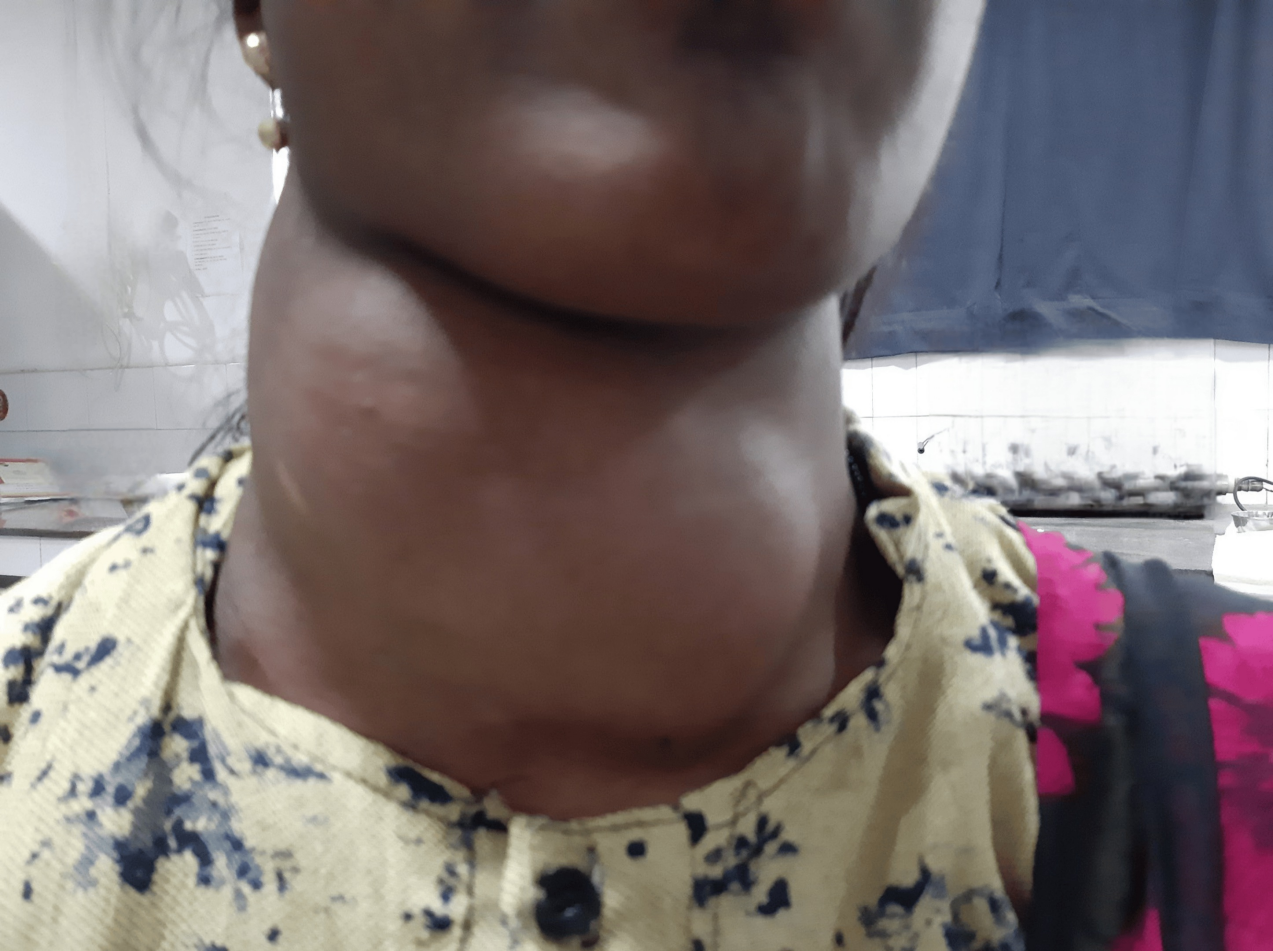
A 25-year-old-lady with thyroid malignancy.

A total of 32 patients were included in the study, with 87.5% (n=28) being female and 12.5% (n=4) being male (Figure 2). The mean age was 44.375 + 12.09 years.

The most common diagnosis was benign multinodular goiter (MNG), representing 43.8% (n=14) of the cases, followed by papillary carcinoma at 37.5% (n=12). follicular neoplasm and lymphocytic thyroiditis were each diagnosed in 6.3% (n=2) of patients, while Hürthle cell carcinoma and medullary carcinoma were observed in one person each. Hence 56.3% (n=18) cases were benign, and 43.8% (n=14) cases were malignant. Tracheal deviation to the right was noted in 34.38% (n=11) cases, tracheal deviation to the left was noted in 28.12% (n=9) and centrally placed trachea was noted in 37.4% (n=12) cases. All cases underwent surgery through a cervical incision and sternotomy was not required in any of the cases. Five patients had tracheomalacia of which three underwent tracheostomy. Two patients presented with stridor and underwent tracheostomy preoperatively. Among them, one patient had tracheal compression, which caused the narrowing of the airway. The other patient had stridor resulting from vocal cord palsy, leading to airway obstruction (Table 1).

**Table 1:** General characteristics and tracheal status.

Variables		Count (n)	%
Gender	Female	28	87.50%
	Male	4	12.50%
Diagnosis	Follicular neoplasm	2	6.30%
	Hürthle cell carcinoma	1	3.10%
	Lymphocytic thyroiditis	2	6.30%
	Medullary carcinoma	1	3.10%
	Benign multinodular goiter	14	43.80%
	Papillary carcinoma	12	37.50%
Position of trachea	Central	12	37.40%
	Deviated to right	11	34.38%
	Deviated to left	9	28.12%
Tracheal compression	Anteroposterior	8	25.00%
	Lateral	6	18.80%
Mode of intubation	Direct laryngoscopy	11	34.38%
	Video laryngoscopy	10	31.25%
	Flexible fiberoptic-assisted intubation	9	28.13%
	Preoperative tracheostomy	2	6.25%

The study included 32 patients, divided into two groups: benign (n=18) and malignant (n=14). The prevalence of dyspnoea was higher in malignant cases (35.71%, n=5) compared to benign cases (16.67%, n=3), though the difference was not statistically significant (p=0.108). Similarly, dysphagia occurred in 16.7% (n=3) of benign cases and 14.3% (n=2) of malignant cases, with no significant difference (p=0.854). Preoperative hoarseness was present in one benign case and three malignant cases (21.4%), but again, the difference was not significant (p=0.174). Two patients presented with stridor and had malignant disease.

The tracheal deviation was present in 78.6% (n=11) of malignant cases and 50% (n=9) of benign cases (p=0.0488), while anteroposterior tracheal compression was seen in 42.9% (n=6) of malignant cases and 11.1% (n=2) of benign cases (p=0.047) which was statistically significant. Lateral tracheal compression was more frequent in malignant cases (28.6%, n=4) compared to benign cases (11.1%, n=2), but the difference was not statistically significant (p=0.1785). One patient had minimal infiltration of the anterior tracheal wall for which sleeve resection of the tracheal rings with repair of the tracheal wall was done.

**Figure 3:**
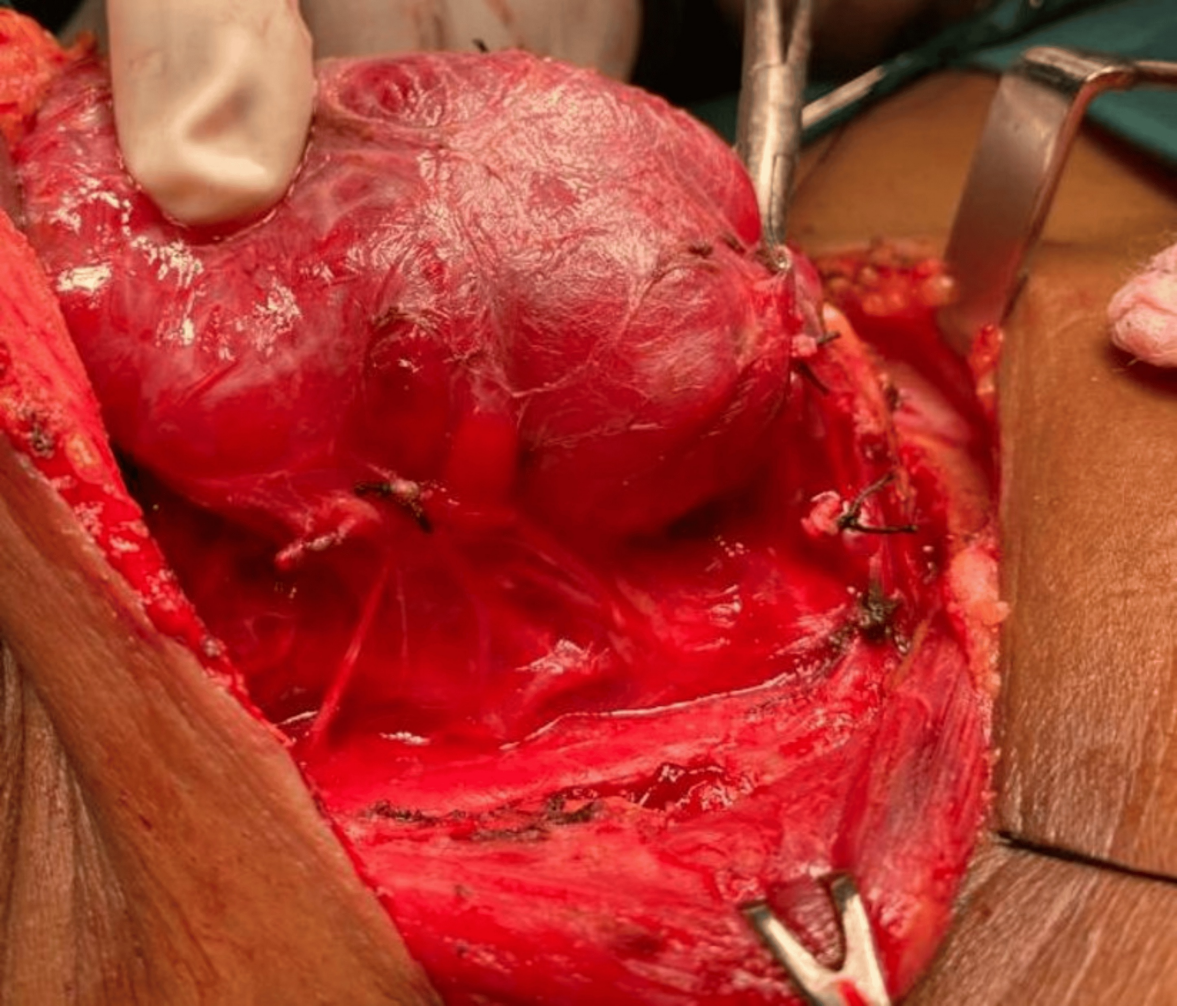
Total thyroidectomy in a 50-year-old lady with thyroid malignancy.

In this study, the extent of the retrosternal extension was primarily less than 1 inch (46.87%, n=15), with few cases extending between 1-2 inches (31.25%, n=10) and more than 2 inches (21.88%, n=7). Lymph node involvement was most common in lateral neck nodes- levels II, III, IV (34.36%, n=11), followed by a combination of lateral and central compartment nodes (21.88%,n=7), only central compartment nodes (15.63%, n=5) and mediastinal lymph nodes (6.25%, n=2). The majority of surgeries performed were total thyroidectomies (TT) at 50.00% (n=16), with fewer undergoing TT combined with central compartment clearance (CCC) (40.63%, n=13), total thyroidectomy with central compartment and mediastinal lymph node clearance with modified radical neck dissection (3.13%, n=1) and total thyroidectomy with central compartment and mediastinal lymph node clearance, radical neck dissection and modified radical neck dissection (6.25%, n=2) (Figure 3). Blood loss during surgery was less than 200 ml (40.62%) in the majority of cases (Table 2).

**Table 2:** Intra-operative details. TT: total thyroidectomy, CCC: central compartment clearance, MC: mediastinal clearance, MRND: modified radical neck dissection, RND: radical neck dissection.

Variables		Count (N)	%	
Extent of retrosternal extension	<1 inch	15	46.87%	
	1-2 inches	10	31.25%	
	>2 inches	7	21.88%	
Lymph nodes	Central compartment nodes	5	15.63%	
	Mediastinal lymph nodes	2	6.25%	
	Lateral neck nodes (Levels II, III, IV)	11	34.36%	
	Lateral and central compartment nodes	7	21.88%	
				Surgery performed	TT	16	50.00%	
TT + CCC	13	40.63%		
TT + CCC + MC + MRND	1	3.13%		
TT + CCC + MC + MRND + RND	2	6.25%		
Duration of surgery	>3 hours	4	12.50%	
	>4 hours	11	34.38%	
	>5 hours	6	18.75%	
	>6 hours	11	34.38%	
Blood loss	<100 ml	4	12.50%	
	<200 ml	13	40.62%	
	<300 ml	10	31.25%	
	<400 ml	3	9.38%	
	<500 ml	2	6.25%	

Postoperative complications included hypocalcemia in 43.80% (n=14) of cases, along with hematoma in the neck and recurrent laryngeal nerve palsy, each at 6.25% (n=2). The average length of hospital stay was between five to 10 days (40.63%, n=13) in the majority of cases, which was extended due to the need for correction of hypocalcemia in the postoperative period (Table 3).

**Table 3:** Postoperative complications and hospitalization.

Variables		Count (n)	%
Tracheomalacia		5	15.63%
Duration of postoperative ventilator dependency	2 days	3	9.38%
	3 days	1	3.13%
Postoperative complications	Hematoma	2	6.25%
	Recurrent laryngeal nerve palsy	2	6.25%
	Hypocalcemia	14	43.80%
Duration of hospital stay	<5 days	9	28.13%
	5-10 days	13	40.63%
	10-15 days	6	18.75%
	>15 days	4	12.50%

Of the total patients, 62.6% (n=20) had tracheal deviation, while 37.4% (n=12) had a central trachea. Dyspnoea was present in 62.5% (n=5) of patients with anteroposterior compression, 16.67% (n=1) of patients with lateral compression, and 11.11% (n=2) of patients with no tracheal compression with a p-value of 0.0176, indicating a statistically significant correlation between tracheal compression and dyspnoea. The duration of swelling was significantly associated with tracheal deviation (p=0.0366), where patients with less than one year of swelling were more likely to have a central trachea (58.33%) compared to those with tracheal deviation (15%). However, no significant association was observed between swelling duration and tracheal compression (p=0.7816).

Postoperative ventilator dependency was required in 15% (n=3) of patients with tracheal deviation and 8.33% (n=1) of patients with a central trachea (p=0.027). Among patients with anteroposterior compression, 37.5% (n=3) required elective intubation, whereas none of the non-compressed patients required it (p=0.0268).

Postoperative complications were documented, with 8.33% (n=1) of both central and deviated trachea patients experiencing hematoma, though this was not statistically significant (p=0.677). RLN palsy was noted in 10% (n=2) of patients with tracheal deviation but absent in central trachea cases. Hypocalcemia was observed in 50% (n=6) of patients with central trachea and 40% (n=8) with tracheal deviation. Among patients with anteroposterior compression, 62.5% (n=5) had hypocalcemia, while only 27.78% (n=5) of non-compressed patients developed this complication (Table 4).

**Table 4:** Comparative analysis of tracheal deviation and compression on clinical symptoms and postoperative outcomes. RLN palsy: recurrent laryngeal nerve palsy.

Variables		Tracheal deviation						Tracheal compression							
		Central (12)		Deviated (20)		p value	Chi-square value	Anteroposterior compression (8)		Lateral compression (6)		Not compressed (18)		p value	Chi-square value
		n	%	n	%			n	%	n	%	n	%		
Dyspnea		3	25%	5	25%	1	0.0	5	62.5%	1	16.67%	2	11.11%	0.0176	8.074
Dysphagia		3	25%	2	10%	0.258	1.28	1	12.5%	1	16.67%	2	11.11%	0.9385	0.127
Preoperative hoarseness		0	0	4	20%	0.0977	2.743	2	25%	1	16.67%	1	5.56%	0.3621	2.031
Stridor		1	8.33%	1	5%	1	0.0	1	12.5%	1	16.67%	0	0	0.241	2.844
Duration of swelling	<1 year	7	58.33%	3	15%	0.0366	6.6133	3	37.5%	2	33.33%	3	16.67%	0.7816	1.75
	1-3 years	3	25%	9	45%			3	37.5%	3	50%	10	55.56%		
	4-7 years	2	16.67%	8	40%			2	25%	1	16.67%	5	27.78%		
Intubation	Direct laryngoscopy	4	33.33%	7	35%	0.904	0.565	1	12.5%	1	16.67%	9	50.00%	0.0524	12.462
	Video laryngoscopy	3	25%	7	35%			2	25%	1	16.67%	7	38.89%		
	Flexible fiberoptic-assisted intubation	4	33.33%	5	25%			4	50%	4	66.67%	1	5.5%		
	Preoperative tracheostomy	1	8.33%	1	5%			1	12.5%	0	0	1	5.56%		
Postoperative ventilator dependency		1	8.33%	3	15%	0.027	4.891	3	37.5%	1	16.67%	0	0	0.0268	7.238
Postoperative complications	Hematoma	1	8.33%	1	5%	0.677	1.524	1	12.5%	1	16.67%	0	0	0.0908	10.921
	RLN palsy	0	0	2	10%			1	12.5%	0	0	1	5.56%		
	Hypocalcemia	5	41.67%	9	45%			5	62.5%	4	66.67%	5	27.78%		

## Discussion

Large goiters with retrosternal extension are often associated with multiple complications. The large size of these goiters tends to distort the anatomy of the neck, increasing the risk of injury to important structures like the recurrent laryngeal nerve. The prolonged duration of goiter tends to compress the underlying structures, such as the trachea and the esophagus, leading to symptoms such as dyspnoea and dysphagia. Symptoms of airway obstruction may range from dyspnea to respiratory distress. The prolonged duration of compression over the trachea weakens the tracheal cartilages resulting in tracheomalacia. Following thyroidectomy in these patients, it can cause a collapse of the tracheal wall post-extubation, which can be life-threatening. These factors make surgery and airway management more challenging and result in a higher incidence of complications such as hypocalcemia, recurrent laryngeal nerve injury, and tracheomalacia. Preoperative imaging, such as CT scans, is essential to assess airway compression. However, these scans may not effectively reflect the dynamic variations in airway diameter and may require evaluation by awake fiberoptic bronchoscopy [6].

In our study, of the total 32 cases, 56.3% were benign and 43.8% were malignant. Malignant goiters were associated with a higher incidence of tracheal deviation and compression, causing variable levels of respiratory symptoms from dyspnoea to stridor. These were comparable to the study conducted by Agarwal et al. [1]. They also present a higher risk of hoarseness due to infiltration or compression of the recurrent laryngeal nerve. These airway complications were associated with increased frequency of tracheostomy and prolonged ventilatory support [1].

The preoperative and postoperative management of the airway is critical in retrosternal goiters. In our study, we found that 62.6% of patients had tracheal deviation, 25% of patients had anteroposterior tracheal compression, and 18.8% of patients had lateral tracheal compression. It was observed that the likelihood of tracheal deviation increased as the duration of goiter increased. Patients with tracheal compression had a higher tendency for postoperative complications. Although there was no statistically significant difference, our findings align with previous studies, highlighting the heightened risk of complications and the importance of employing meticulous surgical techniques. Shen et al. reported tracheal deviation in 70% of patients and tracheal compression in 35% of patients. They also noted that tracheal compression was associated with postoperative airway complications in terms of hematoma formation and prolonged ventilatory support, which is similar to our study. However, they did not find any association with tracheal deviation and prolonged ventilatory support [4].

The choice of intubation technique is crucial, especially in cases with significant tracheal narrowing. Though awake fiberoptic intubation is considered the gold standard for anticipated difficult airways, we observed that despite the altered anatomy, most patients were successfully intubated by the use of a Macintosh or a video laryngoscope. Only 28.13% of patients required advanced airway management by a flexible fiberoptic-assisted intubation and two patients underwent preoperative tracheostomy. This observation is consistent with a similar study conducted by Cappellacci et al. and Zuo et al. [6,7]. A study by Tasche et al. found that most patients could be intubated using standard techniques, but those with larger goiters and higher BMI had difficult intubation [8]. In contrast, our study found that tracheal compression itself was a key factor in the need for advanced airway techniques, independent of patient characteristics like BMI. Therefore, it is important that every institution operating such cases should have a fiberoptic endoscope for guided intubation. In cases where intubation is not possible, placing a tube just above the critical compression and ventilating may give us enough time to dissect the gland. Often cutting the strap muscles will release the compression partially on the table. Thus, detailed preoperative evaluation and tailored anesthesia strategies are vital for ensuring safety, especially in instances with significant tracheal compression.

We found that patients who required prolonged ventilation following surgery had tracheal compression. Tracheal deviation and compression were found to be significantly associated with the need for prolonged ventilatory support; 15.63% were found to have tracheomalacia and were managed by tracheostomy and prolonged ventilatory support. In contrast to our study, Agarwal et al. found a higher incidence of tracheomalacia (35.6%) in retrosternal goiters but was managed in a similar manner [1]. Other studies by Sulaiman et al. and Wong et al. reported little to no incidence of tracheomalacia with rates as low as 0 and 7.5% respectively [3,9]. The presence of tracheomalacia requires vigilant monitoring both during and after surgery. After extubation, the risk of airway compromise must be carefully considered, as patients may develop stridor that necessitates immediate intervention, such as reintubation or tracheostomy. Therefore, a multidisciplinary approach to airway management is essential for cases involving tracheal deviation and compression.

In our study, postoperative hypocalcemia was seen in 43.8% of patients, hematoma in 6.25% of patients, and recurrent laryngeal nerve injury in 6.25% of patients. The inferior parathyroid gland is at greater risk of devascularization during mobilization of the gland due to its location near the thyrothymic ligament [4]. Obadiel et al. found an incidence of 31.9% hypocalcemia, 2% hematoma, and 11.6% recurrent laryngeal nerve injury [5]. Most cases were located in the anterior mediastinum, hence the likelihood of injury to the recurrent laryngeal nerve, which is situated in the tracheoesophageal groove, is low. However posterior extensions which alter the path of the nerve may present a greater risk of injury [9]. In a study of 70 patients who underwent total thyroidectomy for retrosternal extension, postoperative complications were noted in 31.9% of patients. Among these patients, hypocalcemia was the most common complication followed by transient hoarseness, postoperative hematoma, and local infection [10,11]. Surgical management of these goiters is more complex and requires a longer operative time due to additional procedures such as central compartment clearance. Despite these complexities, the rate of complications like hypocalcemia and recurrent laryngeal nerve palsy were found to be similar between benign and malignant goiters, highlighting the importance of meticulous surgical techniques to minimize risks [1].

The advent of new technologies such as intraoperative recurrent laryngeal nerve monitoring can help reduce the incidence of injury to recurrent laryngeal nerve [12]. Intraoperative infrared imaging following Indocyanine green fluorescence for identification of the parathyroid gland can further help in preventing postoperative hypocalcemia [13].

Our study highlights the need for a collaborative approach in the management of retrosternal goiters, especially when tracheal compression and deviation are present. Preoperative imaging such as CT scans, and flexible bronchoscopy, play an important role in evaluating airway compromise and preparing for potential complications. Perioperative decisions, including a selection of intubation methods, and the necessity of tracheostomy, should be decided by a multi-specialty team approach. Additionally, careful postoperative monitoring for complications such as airway obstruction, hematoma, and hypocalcemia is essential to facilitate timely intervention and minimize morbidity.

## Conclusions

Accurate preoperative evaluation of goiters with retrosternal extension, to assess the tracheal status in order to predict airway complications in the perioperative period especially in malignant cases is mandatory before treatment. Effective management requires a multidisciplinary approach involving surgeons and anesthesiologists, tailored anesthesia techniques, and may necessitate advanced airway management methods in cases with significant tracheal compression. Despite the complexities associated with these cases, the surgical outcomes showed similar complication rates between benign and malignant cases, underlining the necessity of meticulous surgical techniques to minimize risks. Now with the availability of better technology intraoperative recurrent laryngeal nerve monitoring and infrared imaging following indocyanine green fluorescence of parathyroids may further help in surgery. In large retrosternal goiter, future prospective multi-institutional studies with a larger cohort are necessary to identify the exact incidence of problems and devise effective guidelines.
